# Effects of Early and Late Time-Restricted Feeding on Parameters of Metabolic Health: An Explorative Literature Assessment

**DOI:** 10.3390/nu16111721

**Published:** 2024-05-31

**Authors:** Froso Petridi, Jan M. W. Geurts, Jean Nyakayiru, Anne Schaafsma, Dedmer Schaafsma, Ruth C. R. Meex, Cécile M. Singh-Povel

**Affiliations:** 1 Division of Human Nutrition and Health, Wageningen University and Research, P.O. Box 17, 6700 AA Wageningen, The Netherlands; 2 FrieslandCampina, 3818 LE Amersfoort, The Netherlands; 3 Science Impact, Winnipeg, MB R2J 2L8, Canada; 4 NUTRIM School of Nutrition and Translational Research in Metabolism, Department of Human Biology, Maastricht University Medical Centre+, 6229 ER Maastricht, The Netherlands

**Keywords:** time-restricted feeding, TRF, TRE, chronotype, metabolic health, overweight, weight loss, insulin resistance, randomized controlled trial

## Abstract

Chrono-nutrition (meal timing) aligns food consumption with one’s circadian rhythm. The first meal (e.g., breakfast) likely promotes synchronization of peripheral circadian clocks, thereby supporting metabolic health. Time-restricted feeding (TRF) has been shown to reduce body weight (BW) and/or improve cardiovascular biomarkers. In this explorative literature assessment, 13 TRF randomized controlled trials (RCTs) were selected from PubMed and Scopus to evaluate the effects of early (eTRF: first meal before 10:30 a.m.) and late TRF (lTRF: first meal after 11:30 a.m.) on parameters of metabolic health. Although distinct variations in study design were evident between reports, TRF consistently decreased energy intake (EI) and BW, and improved insulin resistance as well as systolic blood pressure. eTRF seemed to have a greater beneficial effect than lTRF on insulin resistance (HOMA-IR). Importantly, most studies did not appear to consider chronotype in their evaluation, which may have underestimated TRF effects. TRF intervention may be a promising approach for risk reduction of human metabolic diseases. To conclusively determine benefits of TRF and identify clear differences between eTRF and lTRF, future studies should be longer-term (≥8 weeks) with well-defined (differences in) feeding windows, include participants chronotypically matching the intervention, and compare outcomes to those of control groups without any dietary limitations.

## 1. Introduction

Metabolic health is a term indicating how well the body converts food into energy and manages nutrients; it is considered ‘good’ when an individual displays ‘normal’ ranges in health markers such as blood pressure, body weight (BW), glucose, insulin, and lipid blood levels. When one or more of these parameters are disturbed, the risk of developing metabolic diseases such as coronary heart disease (CHD), cardiovascular diseases (CVDs), and diabetes mellitus type 2 (T2D) is considerably increased [1].

Several diets have been proposed to prevent or treat CVDs or T2D either directly, by targeting disease-related metabolic pathways, or indirectly through weight loss [2]. Most popular diets designed to promote metabolic health focus on the type and amount of food intake. However, consideration of specific mealtimes, which has emerged as a potential critical factor in improving or maintaining metabolic health, is often ignored [3,4]. In this regard, mechanisms associated with the circadian clock that directly affect metabolic parameters need to be considered.

Chrono-nutrition explores how the interactions between circadian rhythms and nutrition/metabolism impact overall health [5,6,7]. Circadian rhythms are regulated and synced with the natural light/dark cycle by a master clock in the suprachiasmatic nuclei (SCN) of the hypothalamus. The SCN is the main stimulator of peripheral clocks present in various metabolic tissues, including adipose tissue, the liver, and the intestines, exerting its effects via neurohormonal factors. These clocks drive the circadian expression as well as activity of hormones and enzymes involved in metabolism [8], thereby preparing the body for the first meal (e.g., breakfast) [9]. Notably, food intake also plays a role in synchronizing peripheral circadian rhythms with the SCN [10,11]. Eating breakfast increases the expression of clock genes involved in regulating insulin sensitivity, glucose uptake, and energy expenditure. Therefore, early morning breakfast is important for synchronization of central and peripheral clocks as well as promoting metabolic homeostasis [12]. Other circadian hormones, including the sleep hormone melatonin, regulate energy homeostasis via the melanocortin receptor 4 (MC4R) pathway. MC4R is also the target of appetite hormones (e.g., leptin, ghrelin, and PYY) influencing the cephalic phase of satiety [3]. Thus, circadian rhythms are tightly linked to energy homeostasis and hunger sensation.

When considering the SCN as the most important driver of peripheral clocks, it can be envisioned that early and late chronotypes [6] do not exhibit differences in metabolic responses if subjects follow the same meal pattern (breakfast–lunch–dinner) and the light/dark cycles have been adjusted as well. Circadian misalignment is linked to metabolic disturbances and associated illnesses [9]. In line with this notion, studies have shown that obesity and metabolic disorders are more prevalent in shift workers [13,14], and even a short-term circadian misalignment protocol of 3.5 days, induced by a 12 h rapid shift of the behavioral cycle, has been shown to induce insulin resistance in healthy, lean, young adults [15,16].

Breakfast is popularly regarded as the most important meal of the day. This is supported by a systematic review and meta-analysis of observational and longitudinal studies, which showed an increased relative risk of overweight/obesity when breakfast was skipped [17]. Furthermore, a few studies reported that skipping breakfast was linked to increased levels of fasting insulin, total cholesterol, and low-density lipoprotein cholesterol [18,19]. In agreement with these observations, an intervention study in women with obesity showed that consuming the most calories of the day (700 kcal) during breakfast led to greater weight loss as compared to an evening-loaded diet [20]. Conversely, a systematic review of interventional studies indicated that skipping breakfast was associated with a decrease in BW [21]. In addition, a recent crossover study in adults with obesity revealed no differences in BW when the highest caloric meal (608 kcal) was consumed in the morning or evening, although participants expressed higher satiety and the number of leftovers was significantly greater when subjects participated in the morning-loaded arm of the study [22]. Notably, there are indications that breakfast is important for glucose metabolism. Oral glucose tolerance follows a diurnal pattern, typically peaking in the morning (after fasting during sleep) and decreasing in the afternoon [9,16]. Interestingly, this appears independent of the fasting duration [9], suggesting an earlier eating window (including breakfast) can help to achieve better glycemic control than a later eating window (excluding breakfast). Additionally, ghrelin and some other gut peptides follow circadian rhythms, which may explain why hunger responses are not the same at different times of the day, as suggested by some studies [23,24]. Furthermore, the choice of skipping or consuming breakfast has been linked to personality and behavioral characteristics of the individual [7,25].

Intermittent fasting (IF) represents a type of diet that aims to reduce caloric intake by switching between fasting and eating following a regular schedule, with dietary strategies dictating no caloric intake for 12 up to 48 h [26]. The most well-known IF regimens are fasting for 1 or 2 days per week and eating ad libitum during the other days or eating within a specific daily time window varying from 4 to 12 h. The latter is referred to as time-restricted feeding (TRF), which can be subcategorized into early (eTRF; first meal before 9:00 a.m.), delayed (dTRF; first meal between 9:30 and 10:30 a.m.), and late TRF (lTRF; first meal after 11:30 a.m.; thus, typically excluding breakfast) [27,28,29,30,31,32,33]. Although lTRF is seemingly out of sync with the biological clock (e.g., with regards to glucose tolerance), TRF generally appears to be beneficial for metabolic health by reducing BW associated with improved fasting glucose levels, lipid profile, blood pressure (mainly systolic), insulin sensitivity, inflammation, and appetite [28,33,34,35]. It is conceivable that subjects on a TRF regime (either early or late) are consciously trying to manage their diet and probably snack less than those following a habitual (ad libitum) diet. 

In addition to weight loss, other mechanisms may be involved in the positive effects of TRF. For example, blood lipid and glucose levels are stable during fasting, and prolonging the fasting period may extend those benefits [36]. In addition, the metabolic switch from glucose to fatty acid utilization during fasting may contribute to health improvement. More specifically, fasting prompts the body to degrade stored fat into ketone bodies to generate energy [26], which has been associated with BW reduction and insulin sensitivity improvement [35]. Furthermore, TRF has frequently been linked to a reduction in oxidative stress, which could have beneficial implications for a plethora of neurological and other diseases [37].

The ideal time window for TRF to have optimal effects is a much debated topic. Recent studies have primarily focused on eTRF, which combines the benefits of prolonged fasting, as in IF, while being in sync with the rhythmicity of the circadian clock. Sutton et al. (2018) demonstrated that eTRF improved appetite control by reducing the desire to eat in the evening, which indirectly promotes weight reduction. In addition, it promoted insulin sensitivity, improved β-cell function, and reduced blood pressure as well as oxidative stress [38].

This explorative literature assessment intends to elucidate how major metabolic health characteristics are affected by specific (early or late) TRF regimens. The discussed outcomes will provide useful information to address current gaps in study designs to ultimately determine the impact of eating breakfast on TRF efficiency. 

## 2. Materials and Methods

**Figure 1:**
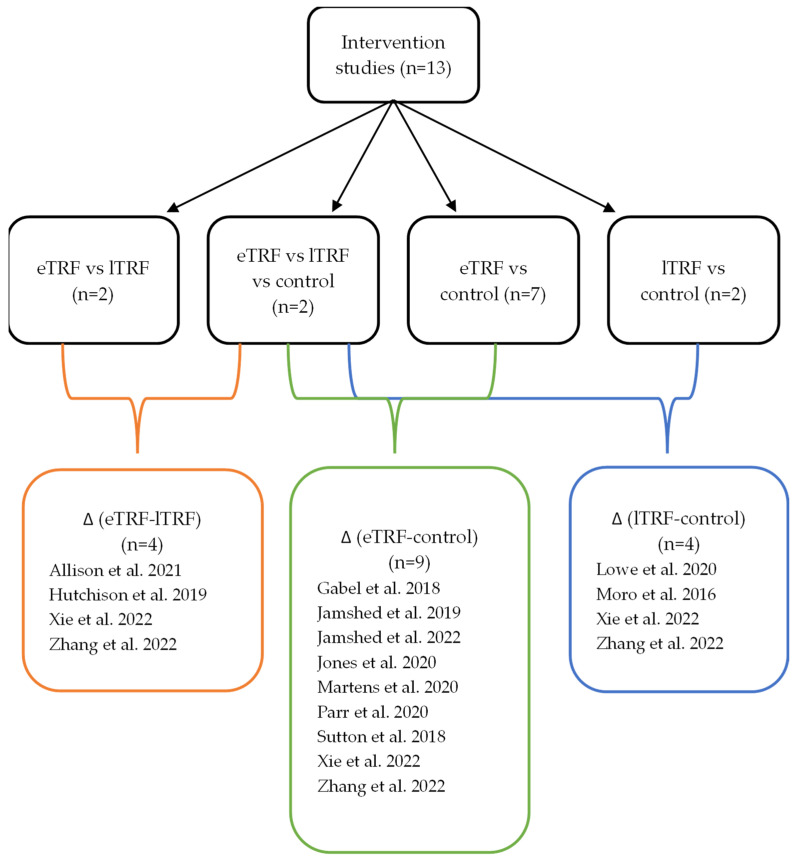
Diagram showing the grouping of study arms and their comparisons (Δ). A total of 13 intervention studies were included. Two studies compared eTRF and lTRF [28,29]; 2 studies compared eTRF, lTRF and control [30,31]; 7 studies compared eTRF and control [34,38,39,40,41,42,43]; and 2 studies compared lTRF and control [32,33]. This resulted in 4 Δ (eTRF-lTRF) [28,29,30,31], 9 Δ (eTRF-control) [30,31,34,38,39,40,41,42,43], and 4 Δ (lTRF-control) [30,31,32,33].

Publications indexed in PubMed and Scopus were searched using combinations of the following keywords: ‘time-restricted feeding’ or ‘TRF’, and ‘breakfast’. Within the databases, the selection criteria ‘adult 19+ years’, ‘clinical trial’, ‘meta-analysis’, ‘RCT’, and ‘(systemic) review’ were used. Studies published before 31 December 2023, were considered. For the present manuscript, only adult human RCT studies providing information on the feeding window (early or late), and investigating metabolic parameters (e.g., BW and glucose metabolism) as primary outcomes were included. References in selected articles were screened for additional studies. No criteria were set for study length, the number of participants included per study arm, or BW class. In eTRF the first meal was consumed between 6:30 and 10:30 a.m. and for lTRF the first meal was consumed after 11:30 a.m. Selected articles were grouped for comparison as follows: eTRF vs. lTRF, eTRF vs. control diets, and lTRF vs. control diets. For differences between groups, effects on specific metabolites were compared (Figure 1). Baseline measurements of a metabolite were subtracted from those after intervention for all studies, except for those of Jamshed et al. (2019) [34], Parr et al. (2022) [39], and Martens et al. (2020) [40], which did not report baseline values. Statistical outcomes were retrieved from the original papers. For specific methods of data analysis, we refer to these reports. Supplementary Table S1 provides an overview of study characteristics and primary outcomes.

## 3. Results

Our search rendered a total of 99 articles: 52 reviews or meta-analyses, 2 observational studies, and 45 RCTs. Thirteen RCTs met the inclusion criteria (Figure 1, Supplementary Table S1), the others were excluded based on the following characteristics: 14 had other primary goals, 5 showed incomplete data, 2 reused data of studies already included, 1 was based on a behavioral outcome, and 10 did not feature specific time windows. After preselection, study arms comprised two (eTRF vs. lTRF), two (eTRF vs. lTRF vs. control), seven (eTRF vs. control), and two (lTRF vs. control) studies. Of note, 11 of the 13 studies included a non-TRF control arm. Based on the four study arms, we created three comparison groups: Δ (eTRF-lTRF) (*n* = 4), Δ (eTRF-control) (*n* = 9), and Δ (lTRF-control) (*n* = 4) (Figure 1).

### 3.1. Study Designs

There was a high degree of variability in study design (weight class, study duration, study parameters) (Table 1 and Table S1). Major variables included the instruction for EI (isocaloric or reduced caloric diet, prescribed diets [28,33,34,38,39,41,42], or habitual/ad libitum food consumption [29,30,31,32,40,43]) and the feeding window of both TRF and control groups. In addition, macronutrient composition differed in the interventional (predetermined) diets between studies, while it was not estimated in ad libitum regimens in most reports [29,30,31,32]. Blood was usually drawn in the morning, although in most studies [28,29,30,34,38,39,40,42] more than one sample per day was collected. The chronotype of participants was essentially not included as a selection criterion. Only three studies used ‘no extreme morning or evening chronotypes’, ‘waking before 9 a.m.’, ‘habitually eating breakfast’, or ‘regular bedtime between 9 p.m. and midnight’ as surrogate markers for chronotype identification [28,39,41]. The 11 studies [30,31,32,33,34,38,39,40,41,42,43] that included a non-TRF control group varied with regards to instructions on study determinants such as food intake, feeding window duration, and first mealtime; however, control groups showed remarkably little variation (Table 1 and Table S1). Because physical activity and coffee consumption were relatively constant between studies, these parameters were considered to not have had a significant impact on study outcomes. Overall, compliance to mealtime (when assessed) ranged from 78 to 100%.

All studies calculated differences between study arms. Some studies additionally calculated the difference of end of study versus baseline per study arm [32,33,39,41]. When a significant difference in metabolic outcomes between study arms was demonstrated, the TRF intervention arm also showed an improvement in metabolic parameters pre- versus post-intervention. In the results section below, only the differences between study arms are presented.

### 3.2. Glucose Metabolism

**Figure 2:**
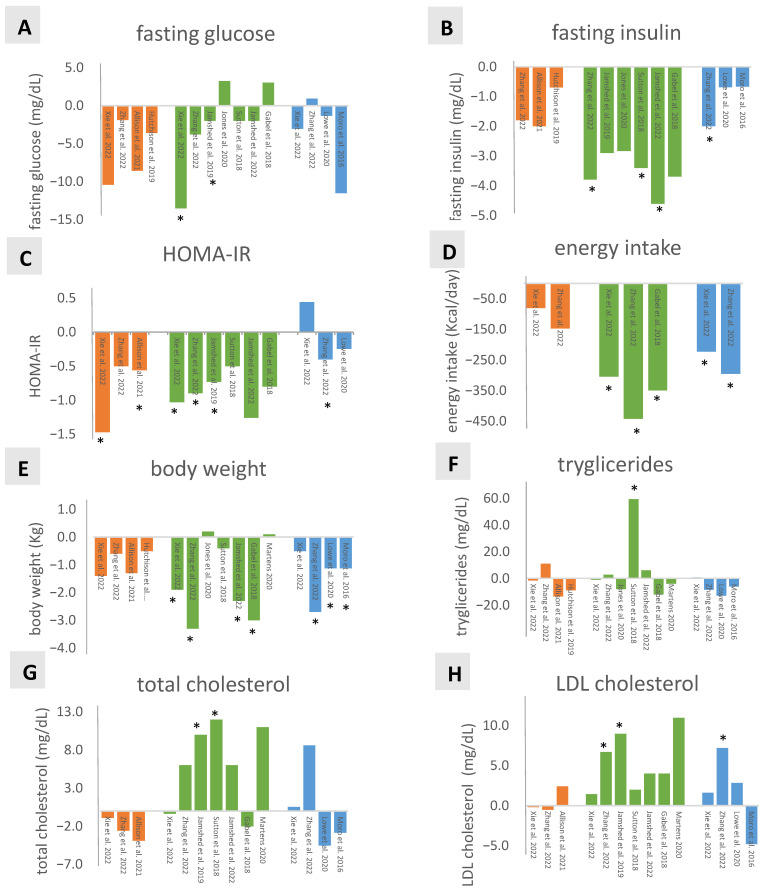

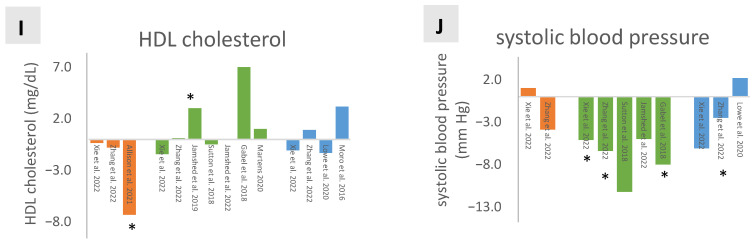
Comparisons (Δ) of the changes in (**A**) fasting glucose levels, (**B**) fasting insulin levels, (**C**) HOMA-IR, (**D**) energy intake, (**E**) body weight, (**F**) triglycerides, (**G**) LDL cholesterol, (**H**) total cholesterol, (**I**) HDL cholesterol, and (**J**) systolic blood pressure between eTRF, lTRF, and/or control groups. Each bar represents the difference between two arms: orange Δ (eTRF-lTRF), green Δ (eTRF-control), and blue Δ (lTRF-control); [number] is the study reference, * *p* < 0.05 [28,29,30,31,32,33,34,38,39,40,41,42,43].

Comparing eTRF-lTRF (*n* = 4) fasting glucose, none of the studies showed a significant difference between groups, although there was a tendency of lower levels in the eTRF compared to the lTRF group (ranging from −10.4 to −1.9 mg/dL; Figure 2A). It is important to note that participants ate ad libitum in three of these eTRF-lTRF studies [29,30,31] and EI was not significantly different in two of these [30,31]. As compared to controls, eTRF significantly decreased fasting glucose in two studies [30,34]. No significant effects were found for lTRF compared to control (Figure 2A). For fasting insulin, eTRF intervention seemed to have a more pronounced, albeit not significant, effect as compared to lTRF (−2 to −0.7 μIU/mL, *n* = 3) (Figure 2B) [28,29,31]. Compared to controls, eTRF significantly decreased fasting insulin in three of six studies (−3.8 to −2.9 μIU/mL) [31,34,38]. The lowering effect of lTRF compared to control on fasting insulin was significant in one of three studies (−1.6 μIU/mL) [31]. In accordance with the effects on fasting glucose and insulin levels, TRF intervention appeared to reduce HOMA-IR (Figure 2C) with eTRF HOMA-IR being lower than lTRF (−1.5 to −0.5, *n* = 3; significant in two studies [28,30]) and controls (−1.26 to −0.70, *n* = 6; significant in three studies [30,31,34]). lTRF showed significantly lower values than controls in one of three studies [31].

A total of six studies reported results on prolonged glucose measurements (iAUC (mg/dL/h)) during the day, assessed by CGM (continuous glucose monitoring) or in venous blood samples [29,31,34,38,39,42]. Two of these studies [34,39] suggested that 24 h glucose iAUC was lower in eTRF than control groups (*p* = 0.003 and *p* = 0.09, respectively). Three studies showed that nocturnal glucose iAUC was significantly lower in eTRF compared to controls, whereas there was no difference at daytime [34,39,42].

### 3.3. Energy Intake and Body Weight

Figure 2D depicts the impact of eTRF and lTRF on EI. EI decreased (−304 to −404 kcal/day) in TRF groups as compared to controls in all three studies in which participants maintained their habitual or ad libitum diets [30,31,43]. However, EI changes were not different between eTRF and lTRF in two of these investigations [30,31]. From four other studies, with ad libitum or habitual diets, information on EI change was lacking [29,32,40,42]. In seven studies, EI was predetermined by investigators: isocaloric diets [28,34,38,39], control group with reduced EI [42], or EI reduction in both study groups [41]. Of the six studies in which both study groups were treated equally, three did not include information on the difference in EI compared to controls [34,38,39]. The other three studies reported no significant differences in EI between TRF and control groups at the end of the investigational period [28,33,41].

With the exception of two studies [34,39], all reports provided information on BW (Figure 2E). When directly comparing eTRF with controls (*n* = 7), four studies reported significantly more pronounced BW reduction in eTRF groups (−3.3 to −1.9 kg) [30,31,41,43], while three studies showed negligible, insignificant effects [(−0.04 kg) [38] and (+0.1 to +0.2 kg) [40,42]]. Three of four lTRF-control comparisons indicated a significantly stronger reduction in BW of participants on lTRF diets (−2.7 to −1.1 kg) [31,32,33]. The apparent tendency of a stronger BW decrease in eTRF compared to lTRF (−0.5 to −1.4 kg, *n* = 4) was not significant.

### 3.4. Lipid Profile

Overall, changes in lipid profile were limited and inconsistent. One of 11 studies reported a significant increase in fasting TG (57 ± 13 mg/dL) when comparing eTRF to controls after 5 weeks of intervention (Figure 2F), which is possibly due to the longer fasting duration preceding testing (18 h versus 12 h in the control arm) [38]. Total cholesterol was not significantly different between eTRF and lTRF, or lTRF and control groups in most of the studies [28,30,31,32,33,34,38,41,43]. Only Jamshed et al. [34] showed a significant increase in total cholesterol (10 mg/dL) in the eTRF as compared to the control group. Sutton et al. [38] found a significant decrease in the control group (−12.8 mg/dL) with no change in the eTRF group (Figure 2H). LDL cholesterol increased in two of seven eTRF studies compared to control groups (Δ eTRF − Δ Control 10–12 mg/dL), and in one of four lTRF studies as compared to controls [31,34] (Figure 2G). One of three studies (Figure 2I) observed a significant decrease in HDL cholesterol (−7.3 mg/dL) when comparing eTRF with lTRF [28], while another study reported a significant increase (3 mg/dL) when directly comparing eTRF with controls [34].

### 3.5. Systolic Blood Pressure (SBP)

SBP was measured in five studies [30,31,32,38,41,43] (Figure 2J). Direct comparison of eTRF and lTRF revealed no differences. Three of five studies comparing eTRF with controls showed a significant decrease in SBP in the intervention group (−6.4–11.2 mm Hg) [31,38,43]. One study showed a significant decrease in SBP (−2.5 mm Hg) in the lTRF compared to the control group [31].

### 3.6. Hunger Sensation

In 6 of 13 studies [29,30,31,38,39,40], participants were asked about feelings of hunger/satiety. eTRF reduced hunger over the course of the day in four of these investigations [31,38,39,40], whereas two studies reported no effects between eTRF, lTRF, and/or control groups [29,30].

## 4. Discussion

With this explorative literature assessment, highlighting 13 relevant RCTs, we aimed to gain a better understanding of the effects of TRF, more specifically eTRF and lTRF, on parameters of metabolic health. As compared to control groups (non TRF), TRF (both early and late) generally decreased EI and reduced BW. In addition, TRF likely improved glucose homeostasis, insulin sensitivity, and SBP. Notably, eTRF may have additional benefits over lTRF, considering its effects on insulin resistance (HOMA-IR) were significantly more pronounced in two of the three studies that directly compared eTRF and lTRF. The effectiveness of eTRF on HOMA-IR may further be supported by findings from investigations in which eTRF was compared with controls, showing significant beneficial effects in three out of six studies.

The lack of other or clearer differences between eTRF and lTRF may be due to the limited number of included studies. Furthermore, there are distinct variations in study design, such as study population, starting BMI, fasting glucose levels, feeding window, and intervention duration (Table 1 and Table S1). For instance, in three of nine eTRF vs. control studies, control diets were isocaloric or had an energy restriction in populations with overweight (based on mean BMI), which likely resulted in BW reduction, thereby benefitting insulin, glucose metabolism, and possibly SBP. Additionally, study duration may have been too short in some studies to detect significant effects. For instance, a recent review reported a 3–5% weight loss as a result of TRF over the course of 2–12 months, possibly peaking around 3 months [44]. This might indicate that in a healthy population, the effects of eTRF on glucose and insulin levels, which are related to BW changes, occur gradually [28]. Nevertheless, such an extended interventional period was only achieved in three of the reviewed studies [32,41,43]. Furthermore, the level of physical activity likely impacts the outcomes of nutritional intervention studies. However, all studies in which physical activity was evaluated [28,29,31,34,39,43] reported no differences in physical activity between study groups. Finally, in three eTRF vs. control studies, eTRF started at 10 a.m., which is relatively late for people who are used to having their first meal at around 8 a.m. In those cases, eating at 10 a.m. might be too late to fully benefit from the effects of the biological clock on insulin and glucose metabolism [3]. Altogether, these factors may lead to an underestimation of the effects of eTRF versus lTRF on metabolic health.

A decrease in EI as a result of TRF might be due to reduced hunger feelings as shown in four of six studies reporting on satiety [31,38,39,40]. In addition, the time-restricted period may be too short to eat all the food one would consume when following a non-time-restricted feeding regime. Increased blood ketone levels (βHB levels ≥ 0.3 mmol/L), produced during prolonged fasting, have been shown to reduce hunger, possibly through, among other mechanisms, a decreasing effect on the secretion of ghrelin and an increase in adiponectin [45,46,47,48,49,50,51]. Although TRF diets have been linked to a moderate elevation of ketone bodies, it remains unclear whether these elevated levels are sufficient to affect satiety [34]. The finding that hunger and plasma ghrelin levels are reduced following breakfast consumption [52,53] suggests that eTRF could be more effective than lTRF in increasing satiety [22]. 

BW followed a very similar pattern to EI, with 7 of 11 TRF vs. control studies showing a significant decrease. A recent review also reported a moderate decreasing effect (3–5%) of TRF on BW, based on RCTs (*n* = 8) lasting for at least 1 month, without EI restriction, no exercise intervention, and unrelated to religious fasting. However, chronotype was not included in the evaluation [44]. Four of the reviewed studies were also highlighted in the present study. Although direct comparisons between eTRF and lTRF did not reveal significant differences, decreases with eTRF appeared slightly stronger. Interestingly, BW was not only lower in case of ad libitum (no diet restrictions) food intake, but also in people subjected to isocaloric interventions (early and late TRF versus control). A decrease in BW might also lower SBP; however, the reviewed studies do not show a clear relationship between the magnitude of effects on BW and SBP [38,54,55]. In addition to EI, the increased resting energy expenditure in the morning hours may have contributed to additional weight loss in eTRF [56], possibly caused by the circadian rhythm of thyroid hormones (TSH and T_3_), stimulating thermogenesis in brown adipose tissue, and ATPases in skeletal muscles [57,58]. Prolonged fasting (as is the case with TRF) increases adiponectin synthesis, which is associated with lipid and glucose processing as well as the regulation of part of the circadian rhythm of hepatic metabolism [33,35,59,60,61]. Based on the early chronotype circadian rhythm, adiponectin peaks around 11 a.m. [62]. Three reviewed studies reported on adiponectin, of which two showed increased levels in the lTRF group (lTRF-control, and lTRF-eTRF).

A total of six studies included lean mass as a parameter. No changes were reported in four of these investigations, one study showed a tendency of a decrease in lTRF [32], and one study indicated a significant decrease in eTRF [31]. It must be noted that the eTRF intervention in Zhang et al. [31] was lower in protein as compared to control and lTRF diets.

BW reduction can lead to better glycemic control [44]. In the discussed studies, TRF particularly improved fasting insulin and HOMA-IR (Figure 2C), while the effects on fasting glucose (reported in 12 of the 13 studies) were less clear and only significant in 2 eTRF vs. control studies [30,34]. Of note, most baseline levels of fasting glucose were in the normal range (<100 mg/dL). Two studies had been performed on participants with overweight and prediabetes, with the highest average fasting glucose levels (100 ± 6 and 110 ± 18 mg/dL); these reports also showed only small and insignificant decreases following the intervention as compared to controls [38,41]. Several trials in humans suggested that TRF may be more effective at reducing insulin levels and improving insulin sensitivity than lowering fasting glucose levels (outcomes described and referenced in [38]). Nevertheless, glucose iAUC (reported in six studies) might improve with eTRF, as indicated by a significantly lower 24 h glucose iAUC in one study [34] and lower nocturnal glucose iAUC in three studies [34,39,42]. A recent review on eTRF and glycemic profile effects (featuring four studies also reviewed in the present paper) suggests that in contrast to fasting glucose, eTRF more likely provides positive effects on postprandial glucose levels in individuals with a healthy BMI [63].

Other TRF-related benefits on glucose metabolism, independent of BW reduction, include the production of ketones due to fasting (thereby positively affecting insulin sensitivity) [64,65], a more frequent meal consumption leading to less fluctuation of insulin secretion, and chrono-nutrition-related mechanisms [65]. eTRF may have greater benefits on glucose metabolism compared to lTRF due to mechanisms related to chrono-nutrition. For instance, melatonin inhibits glucose-mediated insulin secretion; its decrease early in the morning is an important chrono-nutrition mechanism leading to higher insulin sensitivity in the morning [3,66,67]. The second meal effect, another possible chrono-nutrition mechanism, suggests that metabolic responses to an initial meal can alter those to subsequent meals. For example, eating breakfast (rich in proteins and fibers) results in better control of the postprandial glucose responses following lunch and dinner [68,69]. Amplified levels of the incretin hormones GIP and GLP-1 in the morning, resulting in faster insulin responses [7], represent another aspect of chrono-nutrition. Breakfast consumption reduces FFA levels during the whole day, which also leads to reduced blood glucose levels [70]. Furthermore, insulin production in and release from pancreatic β-cells are partly regulated by clock proteins (CLOCK and BMAL1) [57]. Taken together, there is a decent body of evidence to support that breakfast facilitates glycemic control.

Based on the RCTs evaluated in this report, the effects of TRF on lipid metabolism appear to be limited and, therefore, remain largely unclear. For most studies, evaluating the lipid profile was not the primary goal. Only one study, on persons with overweight and prediabetes, indicated a significant increase in fasting TG in the eTRF group [38]. It can be hypothesized that the reported effect on TG is due to the extended fasting period (18 h) as compared to controls (12 h) [38]. Fasting leads to a reduced uptake of circulating TG by adipocytes, while the release of stored TG is stimulated [71]. In two studies, eTRF increased total cholesterol [34,38]. Two studies reported that eTRF [31,34] and one study that lTRF increased LDL cholesterol [31], while eTRF decreased or increased HDL cholesterol [28,34]. Most studies, however, found no effect of TRF on lipid levels. Generally, IF has been reported to benefit the blood lipid profile with decreases in cholesterol (total and LDL) and TG [72]. TRF has also been suggested to have beneficial effects on LDL cholesterol and TG [73], although these effects were not reported in TRF studies in adults with a BMI ≥ 25 [74]. In line with the present paper, more recent studies did not show substantial effects of TRF on cholesterol [75], not even in combination with caloric restriction [76]. Clearly, additional research is needed to conclusively evaluate the impact of TRF on cholesterol metabolism. 

This explorative assessment supports the presumed benefits of TRF on BW reduction and insulin resistance. However, due to considerable differences in study design and a relatively small number of reviewed studies, other benefits may have been overlooked. The current work is a non-systematic literature assessment, which means that the results do not necessarily have statistical significance but rather provide a possible foundation for the direction of future studies. Moreover, not all studies evaluated the same metabolites/markers, and a few investigations ignored baseline measurements for some variables, which complicated comparing the observed outcomes between the various RCTs. In addition, not all studies indicated the macronutrient composition of participants’ food intake (both isocaloric and ad libitum) or did so to a limited extent; this is unfortunate considering food composition importantly dictates the metabolic response. Furthermore, some studies did not disclose information on EI; therefore, it could not be validated if interventions were always isocaloric (even if they were meant to be so) or if EI decreased in all studies in which TRF was compared to a habitual diet. Lastly, chronotype, based on habitual waking up before 9 a.m., eating breakfast, or going to bed before midnight, was only considered in three of the reviewed studies. Because of the close link between chronotype and the circadian clock [77,78], a mismatch of chronotype with the intervention (eTRF or lTRF) might affect the initial outcome; for example, when an early bird follows a lTRF regimen.

In order to conclusively determine and compare the effects of eTRF and lTRF on metabolic parameters, future studies should be aimed at evaluating the same set of biomarkers, implementing an intervention duration of at least 8–12 weeks (preferably ≥12 weeks), properly documenting as well as defining meal compositions, and having strict feeding windows that are sufficiently different between eTRF and lTRF interventions. Furthermore, only participants with a normal BMI (or above average depending on the research question) whose chronotypes fit the intervention should be included, without EI restrictions and following their usual physical activity pattern. Information on sleeping patterns and genetics (SNPs and gene expression of circadian rhythm- and/or metabolic health-related genes) may provide useful mechanistic insights to support or explain (unexpected) outcomes [5,10,79].

## 5. Conclusions

This explorative literature assessment suggests that TRF (both early and late) likely decreases EI as well as BW, and could improve satiety, insulin sensitivity, and systolic blood pressure. Effects on fasting glucose were less clear. Regarding the lipid profile, some increased levels were reported for LDL and total cholesterol, although these effects were mostly insignificant. Notably, eTRF showed improved insulin sensitivity as compared to lTRF. The degree of TRF effectiveness will likely be strengthened by adhering to chrono-nutrition principles (feeding in accordance with the chronotype), as well as by optimizing the composition of the first meal (breakfast) to meet the metabolic preferences of the circadian clock. Neither chronotype nor breakfast composition was a major parameter in the reviewed studies. In closing, to validate the specific benefits of TRF in risk reduction of human (metabolic) diseases, there is a need for future studies that implement a longer duration of intervention with clear (differences in) feeding windows, dietary records to assess energy intake and dietary composition, inclusion of participants chronotypically matching the intervention, and comparison of outcomes to a control group without any dietary or time limitations. Furthermore, in addition to studies in the assumed healthy population, future RCTs focusing on the effects of TRF in specific populations (e.g., overweight/obese individuals or T2D patients) should be considered. 

## Figures and Tables

**Table 1:** Variations in study design of the 13 included RCTs.

Total number of studies (*n*)	13
Number of participants per study (*n*)	8 to 90
Ethnicity	All ethnicities (usually >1 in each study)
Age range (years)	22.1 to 68
BMI range (kg/m^2^)	21.4 to 39.6
Fasting glucose baseline (mg/dL)	73.4 to 110
Intervention groups (arms)	2× eTRF vs. lTRF [28,29] 2× eTRF vs. lTRF vs. control [30,31] 7× eTRF vs. control [34,38,39,40,41,42,43] 2× lTRF vs. control [32,33]
Caloric intake	5× isocaloric or energy resricted [33,38,39,41,42] 6× ad libitum/habitual diets [29,30,31,32,40,43] 2× prescribed, isocaloric diets [28,34]
Feeding window (h)	6 to 11 h for TRF and >8 to 15 h for control
Intervention duration	4 days to 14 weeks
Macronutrient composition (%)	Carbohydrates: 45–55% Fat: 23.9–35% Protein: 15–21.4%
Meal frequency	IF predetermined: 3 to 5 meals/day
Breakfast calories (in TRF arms)	33–40% of energy intake

## Supplementary Materials

The following supporting information can be downloaded at: https://www.mdpi.com/article/10.3390/nu16111721/s1, Table S1: Characteristics of the 13 RCTs discussed in this paper.

## Abbreviations

BMAL1	Basic helix-loop-helix ARNT like 1
BW	Body weight
CLOCK	Circadian locomotor output cycles kaput
CVD	Cardiovascular disease
EI	Energy intake
eTRF	Early time-restricted feeding
GIP	Gastric inhibitory polypeptide
GLP-1	Glucagon-like peptide-1
HDL	High density lipoprotein
HOMA-IR	Homeostatic model assessment for insulin resistance
iAUC	Incremental area under the curve
IF	Intermittent fasting
LDL	Low density lipoprotein
lTRF	Late time-restricted feeding
MC4R	Melanocortin receptor 4
PYY	Peptide YY
RCT	Randomized controlled trial
SBP	Systolic blood pressure
SCN	Suprachiasmatic nuclei
SNP	Single nucleotide polymorphism
T2D	Type 2 diabetes mellitus
TG	Triglycerides
