# Supplementary material for: Effects of Early and Late Time-Restricted Feeding on Parameters of Metabolic Health: An Explorative Literature Assessment
Source: Nutrients. 2024 May 31;16(11):1721. doi: 10.3390/nu16111721 (PMC11175017; doi:10.3390/nu16111721)
Supplement: Supplementary file 1 [file nutrients-16-01721-s001.zip › nutrients-3004253-supplementary.pdf]

Supplementary Table S1: Characteristics of the 13 RCTs discussed in this paper.

Source	Participants	Type of study	Length (week)	Feeding window	Group	Baseline values: Fasting glucose (mg/dL)	Baseline values: Fasting insulin (µIU/ml	Baseline values: Body weight (kg)	Change (end-baseline): Fasting glucose (mg/dL)	Change (end-baseline): Fasting insulin (µIU/ml	Change (end-baseline): Body weight (kg)	Outcomes
**Allison 2021 [28]**	12 (5F) Healthy to prediabetic. BMI: 21.9 ±1.7 kg/m^2 Age: 26.3 ± 3.4 y Isocaloric diets	Cross-over	8 wk., 2 wk. washout	8am - 7pm	eTRF	95.3	8.1	65.4	-3.7	-1.0	-1.1	When comparing eTRF to lTRF, eTRF significantly improved insulin resistance (HOMA-IR) and trunk-to-leg fat ratio, while lTRF ameliorated dHDL cholesterol. eTRF tended to improve BW, fat oxidation, fasting glucose, insulin, triglycerides, and total cholesterol, while lTRF seemed to better LDL cholesterol, adiponectin, and resting energy expenditure.
				12pm - 11pm	lTRF	92.1	6.8	64.8	4.9	1.0	0.2	
**Hutchison 2019 [29]**	15 (0F) Prediabetic BMI: 33.9 ± 0.8 Age: 55.0 ± 3 Test drinks on test days	Cross-over	1 wk., 2 wk. washout	8am - 5pm	eTRF	104.4	9.5	105.7 ± 2.6	-1.8	-1.12	-1.3 ± 0.3	As compared to baseline, eTRF significantly lowered fasting glucose. As such, TRF improved glucose tolerance and fasting triglycerides. Fasting ghrelin was lower at 8am versus 12pm, but TRF was without effect.
				12pm - 9pm	lTRF	100.8	8.0		1.8	-0,4	-0.8 ± 0.2	
**Xie 2022 [30]**	28 (24F) BMI: 22.7 ± 3.1 Age: 28.7 ± 9.7	Parallel	5 wk.	6am - 3pm	eTRF			61.1 ± 8.8	-10.6		-1.6	eTRF was more effective than lTRF in improving insulin sensitivity. Furthermore, eTRF, but not lTRF, improved fasting glucose, reduced BW as well as adiposity, ameliorated inflammation, and increased gut microbial diversity. TRF showed lower meal frequency than the control group.
	26 (19F) BMI: 21.4 ± 2.2 Age: 31.1 ± 8.4			11am - 8pm	lTRF			61.0 ± 11.7	-0.2		-0.2	
	28 (21F) BMI: 21.5 ± 2.9 Age: 33.6 ± 11.6			No restriction	Contr			61.2 ± 9.9	2.9		0.3	
**Zhang 2022 [31]**	21(12F) BMI: 27.1 ± 0.7 Age: 23.8 ± 0.6	Parallel	8 wk.	7am - 1pm	eTRF	82.3 ± 1.4	12.4 ± 1.6	78.0 ± 2.9	1.5	-3.4	-3.5	eTRF and lTRF reduced BW as compared to controls. Additionally, eTRF reduced systolic blood pressure, mean fasting insulin, insulin resistance, leptin, and thyroid axis activity, whereas lTRF only reduced leptin levels and thyroid activity. LDL cholesterol was higher in TRF as compared to control groups. No significant differences were found between eTRF and lTRF interventions.
	20 (11F) BMI: 28.5 ± 0.8 Age: 23.2 ± 0.5			12pm - 6pm	lTRF	82.8 ± 1.9	12.2 ± 1.7	83.5 ± 4.1	3.4	-1.6	-2.9	
	19(10F) BMI: 27.8 ± 0.8 Age: 22.1 ± 0.4			No restriction	Contr	80.6 ± 1.3	10.3 ± 1.6	77.1 ± 3.4	2.5	0.4	-0.2	

Source	Participants	Type of study	Length (week)	Feeding window	Group	Baseline values			Change (end-baseline)			Outcomes
						Fasting glucose (mg/dL)	Fasting insulin (μIU/ml	Body weigh t (kg)	Fasting glucose (mg/dL)	Fasting insulin (μIU/ml	Body weight (kg)	
**Jamshed 2019 [34]**	11 (4F)<br>BMI: 30.1 ± 2.7<br>Age: 32.0 ± 7<br>Isocaloric diets	Cross-over	4 days<br>3.5-5 wk. washout	8am - 2pm	eTRF	92 ± 5			-2.0	-2.9	NA	eTRF significantly improved 24h glucose levels, glycemic excursions, and altered diurnal patterns in cortisol and the expression of several circadian clock genes. In the morning, eTRF increased ketones, cholesterol (total, HDL, and LDL), the stress response/aging gene SIRT1, and the autophagy gene LC3A. In the evening, eTRF tended to increase brain-derived neurotropic factor and augmented the expression of mTOR (growth regulation).
				8am - 8pm	Contr				0.0	0.0	NA	
**Jones 2020 [42]**	8 (0F)<br>BMI: 24.0 ± 1.0<br>Age: 22.0 ± 1	Parallel	2 wk.	8am - 4pm	eTRF	72.5	12.3	73.4 ± 3.0	0.9	-1.8	-1.0	As compared to control, eTRF improved whole-body insulin sensitivity and increased skeletal muscle glucose and branch-chain amino acid uptake. BW loss was not different between groups.
	8 (0F)<br>BMI: 23.8 ± 0.5<br>Age: 24.0 ± 2<br>Energy restriction			No restriction	Contr	74.3	6.2	77.7 ± 4.6	-2.3	1.0	-1,2	
**Sutton 2018 [38]**	8 (0F)<br>BMI: 32.2 ± 4.4<br>Age: 56.0 ± 9.0<br>Isocaloric diets	Cross-over	5 wk.<br>7 wk. washout	6 hr.: between 6:30am - 3pm	eTRF	100.0 ± 6.0	23.4 ± 13.9	100.9 ± 18.9	eTRF vs control - 0.5 ± 0.3	eTRF vs control- 3.4 ± 1.6	-1.4	eTRF improved insulin sensitivity (fasting, mean and peak insulin), beta cell responsiveness, blood pressure, oxidative stress (8-isoprostane), and appetite (PYY). However, total cholesterol and triglycerides increased in the eTRF group.
				12 hr.: from 6:30 - 8:30 onwards	Contr	103.0 ± 9.0	24.0 ± 17.8	101.8 ± 19.6			-1.0	
**Jamshed 2022 [41]**	29 (23F)<br>BMI: 39.6 ± 6.6<br>Age: 44.0 ± 11<br>Prediabetic<br>Energy restriction	Parallel	14 wk.	7am - 3pm	eTRF	110.0 ± 18.0	22.7 ± 17	113.8 ± 21.3	-10.0	-6.9	-6.6	Over a feeding window of ≥12 hours, eTRF was more effective for losing BW than the control diet and showed a trend in losing body fat (secondary analysis). eTRF was more effective in improving diastolic blood pressure and mood. Both groups showed reductions in fasting glucose, insulin, and systolic blood pressure.
	30 (24F)<br>BMI: 39.6 ± 6.6<br>Age: 44.0 ± 11<br>Prediabetic			>12 hr., self-selected	Contr	106.0 ± 15.0	18.2 ± 10.8	102.6 ± 19	-7.0	-1.7	-4.3	

Source	Participants	Type of study	Length (week)	Feeding window	Group	Baseline values			Change (end-baseline)			Outcomes
						Fasting glucose (mg/dL)	Fasting insulin (μIU/ml	Body weigh t (kg)	Fasting glucose (mg/dL)	Fasting insulin (μIU/ml	Body weight (kg)	
	Energy restriction											
**Parr 2020 [39]**	11 (0F)<br>BMI: 32.2 ± 2.0<br>Age: 38.0 ± 5<br>Isocaloric diets	Cross-over	5 days<br>10 days washout	10am - 6pm	eTRF	91.7		103.2 ± 9.3				TRF improved nocturnal glycemic control (AUC glucose) and was positively perceived in overweight/obese men. TRF participants had lower ratings of hunger and prospective food consumption from lunch onwards, compared to a rise prior to the late evening meal in the control group.
				7am - 10pm	Contr	93.9						
**Gabel 2018 [43]**	23 (20F)<br>BMI: 35.0 ± 1.0<br>Age: 50.0 ± 2.0	Parallel	12 wk.	10am - 6pm	eTRF	79.0 ± 4.0	8.3 ± 1.0	95.0 ± 3.0	3.0	-2.6	-3.0	BW (–2.6 ± 0.5%), EI (341 ± 53 kcal/d), and systolic blood pressure (–7 ± 2 mm Hg) decreased in the TRF group relative to controls.
	23 (21F)<br>BMI: 34.0 ± 1.0<br>Age: 48.0 ± 2.0			No restrictions	Contr	87.0 ± 2.0	9.2 ± 1.4	92.0 ± 3.0	0.0	1.1	0.0	
**Martens 2020 [40]**	22 (12F)<br>BMI: 24.8 ± 0.8<br>Age: 67.0 ± 2.0	Cross-over	6 wk.<br>No washout	8 hr.: starting at 10 -11am	eTRF	86.0 ± 3.0		68.7 ± 4.0	-3.0		0.7	TRF reduced sensations of hunger. Functional (endurance) capacity and glucose tolerance were modestly improved. TRF caused a moderate significant increase in total and LDL cholesterol. AUC glucose tended to be lower in the TRF as compared to the control group.
				No restrictions	Contr	90.0 ± 2.0		72.0 ± 4.2	-6.0		-2.7	
**Lowe 2020 [32]**	59 (24F)<br>BMI: 32.9 ± 4.9<br>Age: 46.8 ± 10.8<br>Overweight or obese.	Parallel	12 wk.	12pm - 8pm	lTRF	91.7	12.4	99.3 ± 16.9	-1.1	-0.5	-0.9	There was a significant decrease in BW in the TRF p = 0.01), but not in the control group (p = 0.07).
	57 (22F)<br>BMI: 32.6 ± 3.4<br>Age: 46.1 ± 10.3<br>Overweight or obese.<br>3 structured meals/day			7am - 11pm	Contr	93.9	14.7	99.1 ± 15.1	0.29	0.19	-0.7	
**Moro 2016 [33]**	17 (0F)<br>BMI: 26.5<br>Age: 29.9 ± 4.1	Parallel	8 wk.	1pm - 9pm	lTRF	96.6 ± 5.1	2.78 ± 0.6	83.9 ± 12.8	-10.7	-1.0	-1.0	Fat mass, BW, testosterone levels, and insulin-like growth factor expression decreased in the lTRF group as compared to

Source	Participants	Type of study	Length (week)	Feeding window	Group	Baseline values			Change (end-baseline)			Outcomes
						Fasting glucose (mg/dL)	Fasting insulin (μIU/ml	Body weigh t (kg)	Fasting glucose (mg/dL)	Fasting insulin (μIU/ml	Body weight (kg)	
	Resistance trained. Healthy to prediabetic											controls (p=0.045, p=0.035, p=0.048, p=0.040, respectively), as well as T3. Adiponectin (p=0.001) increased, and blood glucose (p=0.001) and insulin (p=0.03) decreased in the lTRF group only.
	17 (0F) BMI: 27.1 Age: 28.5 ± 3.5. Resistance trained. Healthy to prediabetic. Isocaloric to lTRF			8am - 9pm	Contr	95.2 ± 47.8	2.56 ± 0.5	85.3 ± 13.0	0.81	-0.3	0.16	

Note: data as provided by the articles and/or supplemental materials.
Data: expressed as mean, or mean ± SD or SEM
Abbreviations: AUC = area under the curve, BW = body weight, Contr = control, dHDL = direct high density lipoprotein, eTRF = early time restricted feeding, HOMA-IR = homeostatic model assessment for insulin resistance, LDL = low density lipoprotein, lTRF = late time restricted feeding, PYY = peptide YY, , T3 = triiodothyronine.
